# Corneal Perforation as a Possible Ocular Adverse Event Caused by Cabozantinib: A Clinical Case and Brief Review

**DOI:** 10.3390/jcm14124052

**Published:** 2025-06-08

**Authors:** Carmelo Laface, Luca Scartozzi, Chiara Pisano, Paola Vanella, Antonio Greco, Agostino Salvatore Vaiano, Gianmauro Numico

**Affiliations:** 1 Medical Oncology, AO S. Croce e Carle, 12100 Cuneo, Italy; 2 Institute of Ophthalmology, Santa Croce e Carle Hospital, 12100 Cuneo, Italy

**Keywords:** Cabozantinib, vascular endothelial growth factor receptor, corneal perforation, ocular adverse event, renal cell carcinoma

## Abstract

**Background:** Cabozantinib is a Vascular Endothelial Growth Factor Receptor Tyrosine Kinase Inhibitor (VEGFR-TKI). These drugs are employed as therapy for several malignancies. In detail, Cabozantinib has demonstrated its efficacy against several malignancies. On the other hand, Cabozantinib and other VEGFR-TKIs can be responsible for various adverse events (AEs), in particular hepatic and dermatological AEs. **Methods:** To date, limited data are available in the literature regarding ocular AEs due to therapy with these drugs. In this regard, one case of corneal perforation during treatment with a VEGFR-TKI, Regorafenib, has been reported, while there are no data about Cabozantinib. In this paper, we present another clinical case of corneal perforation in a patient affected by advanced RCC and treated with Cabozantinib as a second-line therapy. The patient started Cabozantinib at the dosage of 60 mg/die although it was necessary to apply some dose reductions because of grade 2 AEs (according to CTCAE v6.0), such as asthenia, diarrhea, dysgeusia, and loss of appetite. **Results:** After approximately 15 months of treatment, the patient began to experience pain and vision loss in the right eye. A diagnosis of corneal perforation was made, followed by medical and surgical treatment. As regards the etiology of this pathology, all other possible causes were excluded, including a history of ocular disease, contact trauma, exposure to damaging agents (e.g., chemical agents and prolonged use of drugs such as topical NSAIDs), infections, or dry eye. Therefore, we hypothesized a correlation with Cabozantinib’s mechanisms of action and paused its administration. **Conclusions:** Cabozantinib may alter the ocular environment due to a lack of or imbalance in growth factors in the tear film, with a reduction in corneal epithelium proliferation. This condition might cause dry eye and a delay in corneal healing. Therefore, particular importance should be placed on ophthalmologic surveillance during treatment with these drugs in patients who develop ocular symptoms. Further in vitro and in vivo studies are necessary to deepen the knowledge about VEGFR-TKI-mediated ocular AEs.

## 1. Introduction

Cabozantinib belongs to the Vascular Endothelial Growth Factor Receptor Tyrosine Kinase Inhibitor (VEGFR-TKI) family, which also includes axitinib, lenvatinib, nintedanib, pazopanib, regorafenib, sorafenib, sunitinib, and vandetanib [1]. These drugs act through the inhibition of multiple protein kinases, including those involved in tumor angiogenesis, oncogenesis, and the tumor microenvironment (TME) [1]. VEGFR-TKIs are employed as therapy for several malignancies [2]. In detail, Cabozantinib has demonstrated its efficacy in the treatment of various cancers such as renal cell carcinoma (RCC), hepatocellular carcinoma (HCC), and medullary thyroid carcinoma (MTC) [3,4,5,6,7].

Clinical trials regarding Cabozantinib have reported various adverse events (AEs) that are common to the VEGFR-TKI family, in particular, hepatic and dermatological side effects [3,4,5]. In the literature, there is a paucity of data regarding ocular AEs due to therapy with VEGFR-TKIs, with the literature only referring to retrospective data. No prospective clinical trial has ever described ocular AEs caused by this drug family [8,9]. As regards corneal perforation, one case was described during treatment with Regorafenib, while no case under Cabozantinib or other VEGFR-TKIs has been described [10]. Given the limited knowledge about such AEs, we deemed it necessary to describe another case of corneal perforation in our patient affected by advanced RCC who was undergoing treatment with Cabozantinib as second-line therapy.

In this paper, we discuss all of the mechanisms of action, the efficacy, and the safety profile of Cabozantinib. Subsequently, we focus on the clinical history of our patient treated with Cabozantinib and the developed AEs during treatment, including corneal perforation. Moreover, we discuss the possible correlation between the mechanisms of action of this drug and the occurrence of this AE.

## 2. Cabozantinib: Mechanisms of Action, Clinical Trials, and Adverse Events

### 2.1. Mechanisms of Action

Cabozantinib is a multi-targeted TKI. It is taken orally and exerts its effect by competitively binding to the ATP-binding site on tyrosine kinase receptors (TKRs). In detail, Cabozantinib fits into this crucial site, preventing ATP from aligning correctly. It forms hydrophobic interactions and hydrogen bonds with key amino acids within the catalytic pocket, ensuring targeted and stable action. Thus, Cabozantinib determines the inhibition of substrate phosphorylation and, consequently, the enzymes’ catalytic activity [11].

The main effect of Cabozantinib corresponds to the inhibition of several TKRs playing a critical role in tumor progression. For instance, the drug inhibits neoangiogenesis by blocking Vascular Endothelial Growth Factor Receptor 2 (VEGFR2); this mechanism interferes with angiogenic signaling by disrupting the cascade that stimulates endothelial cell proliferation and migration. The result is a reduction in nutrients and oxygen supply to the tumor, thereby slowing its growth. Furthermore, Cabozantinib targets the Mesenchymal–Epithelial Transition (MET) receptor, which is activated by the Hepatocyte Growth Factor (HGF) and regulates several processes such as cell survival, proliferation, motility, and invasiveness. In detail, the inhibition of MET autophosphorylation leads to the disruption of different signaling pathways that are essential for tumor development, including those mediated by PI3K/AKT, RAS/RAF/ERK, and STAT. In addition, Cabozantinib can target other TKRs, such as RET, AXL, and c-KIT [12,13]. The inhibition of RET is particularly useful in tumors with activating mutations, like medullary thyroid carcinoma, while blocking c-KIT and AXL inhibits cell proliferation and survival [11].

Another mechanism of action of Cabozantinib regards its influence on the tumor microenvironment (TME). In detail, the inhibition of VEGFR2 determines reduced formation of new blood vessels and blood flow inside the tumor. The consequence is the development of hypoxic conditions that can further modify the TME’s features [12,13]. Moreover, MET inhibition leads to reduced release of cytokines and growth factors, rendering the TME less favorable to tumor growth. Furthermore, reduced activation of the PI3K/AKT and RAS/RAF/ERK pathways leads to a reduction in pro-proliferative and anti-apoptotic signals [11].

Cabozantinib’s ability to target multiple kinases may offer an advantage over single-target agents, as the use of TKIs focused on a single target often causes tumor resistance. Additionally, the selective inhibition of VEGFR may trigger compensatory upregulation of the MET pathway, promoting tumor growth. Therefore, the simultaneous inhibition of the VEGF and MET signaling pathways could prevent MET-driven resistance to single-target inhibitors and provide more effective antitumor activity than inhibiting each pathway individually [11].

### 2.2. Clinical Trials

In Europe, Cabozantinib was approved for the treatment of RCC, HCC, and MTC, although this drug was also tested for other solid tumors, including lung and prostate carcinoma.

#### 2.2.1. Renal Cell Carcinoma

Cabozantinib as a single agent is employed for the treatment of advanced RCC as first-line therapy for patients with intermediate/poor risk in terms of prognosis or in combination with Nivolumab, regardless of the risk group [14]. It can also be employed as second-line therapy in those patients who have previously received VEGF-targeted therapy. These therapeutic indications are derived from the METEOR, CheckMate-9ER, and CABOSUN trials, respectively [5,6,7].

The METEOR study evaluated the efficacy and safety of Cabozantinib in patients with advanced RCC who had previously received treatment with at least one VEGFR-TKI. The aim was to compare Cabozantinib versus (vs.) Everolimus. Patients treated with Cabozantinib had a median progression-free survival (PFS) of 7.4 months vs. 3.9 months in the Everolimus group, with a 42% reduction in the risk of progression (hazard ratio [HR] 0.58, 95% confidence interval [CI] 0.45–0.75; *p* < 0.001). Moreover, Cabozantinib improved overall survival (OS). The OS for the Cabozantinib group was 21.4 months compared to 17.1 months for patients receiving Everolimus, with a 34% reduction in the risk of death (HR 0.66, 95% CI 0.53–0.83; *p* = 0.00026). The objective response rate (ORR) was 21% vs. 5% in the Cabozantinib and Everolimus groups, respectively.

The CABOSUN study assessed the efficacy and safety of Cabozantinib in patients with advanced RCC who had not previously received systemic therapy. The study compared Cabozantinib with Sunitinib. The median PFS for patients treated with Cabozantinib was 8.2 months vs. 5.6 months for those receiving Sunitinib, with a 34% reduction in the risk of progression (adjusted HR 0.66, 95% CI 0.46–0.95; one-sided *p* = 0.012). The median OS for the Cabozantinib group was 26.6 months and 21.2 months for the Sunitinib group. Although the survival benefit was observed, the results did not meet the statistical threshold required to confirm a significant difference in OS. The ORR in the Cabozantinib group was 46%, which was notably higher than the 27% observed with Sunitinib.

The CheckMate 9ER study compared the combination of Nivolumab plus Cabozantinib with Sunitinib as a first-line therapy for patients with advanced RCC. Patients who received the therapeutic combination had a median PFS of 16.6 months, significantly longer than the 8.4 months observed in the Sunitinib group. This translated to a 41% reduction in the risk of disease progression or death for the combination group [HR 0.59; 95% CI 0.49–0.71]. The combination treatment also showed a significant improvement in OS (49.5 vs. 35.5 months; HR 0.70; 95% CI 0.56–0.87). Although the results have yet to mature, they suggest a potential for a 31% reduction in the risk of death for the combination therapy. The ORR was 56% vs. 28% in the combination group and Sunitinib group, respectively.

#### 2.2.2. Hepatocellular Carcinoma

Cabozantinib is employed for the treatment of HCC in those patients who have progressed to Sorafenib.

The CELESTIAL trial [3] tested the efficacy and safety of Cabozantinib vs. a Placebo in patients with advanced HCC who had previously received Sorafenib. Cabozantinib significantly improved PFS compared to the Placebo (5.2 months, vs. 1.9 months; adjusted HR 0.44, 95% CI 0.36–0.52; *p* < 0.0001). Patients treated with Cabozantinib had a median OS of 10.2 months, compared to 8 months for those receiving the Placebo (HR 0.76, 95% CI 0.63–0.92; *p* = 0.005). The ORR for Cabozantinib was 4%, vs. 1% seen in the Placebo group (*p* = 0.009).

#### 2.2.3. Medullary Thyroid Carcinoma

Cabozantinib is employed for the treatment of progressive, unresectable, locally advanced, or metastatic MTC.

The EXAM study [4] was a phase III clinical trial designed to test Cabozantinib vs. a Placebo in patients with progressive, metastatic MTC who had received prior treatment. The results showed that Cabozantinib significantly improved PFS compared to the Placebo (11.2 months vs. 4 months; HR 0.28, 95% CI 0.19–0.40; *p* < 0.001). The ORR in the Cabozantinib group was 28% vs. 0% in the Placebo group (*p* < 0.001). The median OS was 26.6 months in the Cabozantinib group vs. 21.1 months in the Placebo group (HR 0.85, 95% CI 0.64–1.12; *p* = 0.2409). An exploratory analysis of about 65% of patients with known RET M918T mutations showed that the median OS was 44.3 months in the Cabozantinib group and 18.9 months in the Placebo group (HR 0.60, 95% CI 0.38–0.94; *p* = 0.026). This subgroup of patients was also noted to have the longest median PFS and highest ORR.

### 2.3. Adverse Events

Cabozantinib, like other TKIs, is associated with a range of AEs, some of which can be quite common, while others are less frequent but more serious. Many of these AEs are manageable with proper monitoring and dose adjustments [3,4,5,6,7,11].

Diarrhea is one of the most common AEs, which affects around 54–75% of patients. Although most cases are mild, approximately 7–21% of patients experience severe diarrhea (grade 3 or 4). Fatigue is another frequent side effect, which is reported in 40–60% of patients. It can range from mild tiredness to more debilitating fatigue, with 10% of patients experiencing severe levels (grade 3–4). Hypertension is also common, affecting around 37–65% of patients on Cabozantinib. Hypertension becomes severe in 10-20% of patients (grade 3 or 4). Hand–foot syndrome (HFS), also known as palmar–plantar erythrodysesthesia, is another frequent AE, occurring in 45–65% of patients, with 8–17% suffering grade 3–4 AEs. It typically causes pain, redness, and swelling on the palms of the hands and the soles of the feet. Weight loss is observed in 35–60% of patients, with 3–9% of severe grade. Nausea is experienced by 30–50% of patients taking Cabozantinib, though it is generally mild. Nausea becomes severe in about 3–5% of cases (grade 3 or 4). Vomiting occurs in 25–30%, with 1–2% of G3–G4. Hair color changes affect around 35% of individuals on Cabozantinib. Decreased appetite and dysgeusia affect 40–45% of patients, with 2–4% experiencing a severe AE. A potential issue that requires careful monitoring is liver enzyme elevation, which occurs in 22–30% of patients. Although severe liver toxicity (grade 3–4) affects 5–8% of patients, it is crucial for healthcare providers to regularly monitor liver function. Constipation is another side effect, seen in 20–25% of patients. While usually manageable with laxatives or dietary changes, severe constipation (grade 3–4) can occur in a smaller subset of patients. Some patients may experience hypothyroidism. It occurs in about 23–37% of patients and is usually manageable with appropriate thyroid hormone replacement therapy.

Furthermore, there is a series of AEs that occurs in almost 10–25% of patients but usually in a mild grade: dyspepsia, dizziness, dysphonia, hyperglycemia, stomatitis, rash, increased blood creatinine levels, proteinuria, anemia, thrombocytopaenia, neutrophil count decreased, asthenia, abdominal pain, cough, peripheral edema, and ascites.

Rarely, in almost 1–2% of cases, patients may experience hemorrhagic events (usually epistaxis), intestinal perforation, and arterial or venous thromboembolism (blood clots, stroke, heart attack, or pulmonary emboli), which are severe complications.

## 3. Clinical Case

In October 2022, a 78-year-old male patient came to our attention at the Oncology Unit of the “S. Croce and Carle” Hospital in Cuneo (CN), Italy. He was experiencing stabbing pain in the sternum. A total body computed tomography (CT) scan revealed a mass of 3 cm in the right kidney and several metastases involving the lungs, lymph nodes, and bones, including one in the sternal body. A biopsy of the sternum was performed; the histological examination confirmed clear cell renal carcinoma (CCRC).

In accordance with guidelines, in December 2022, the patient started treatment with Pembrolizumab plus Axitinib. In August 2023, the first follow-up CT scan showed a numerical increase in bone and lung metastases. In consideration of disease progression, treatment with Cabozantinib was proposed based on the results of the METEOR trial [5]. The patient started therapy at the dosage of 60 mg/die, although he experienced some grade 2 AEs (according to CTCAE v6.0), including asthenia, diarrhea, dysgeusia, and loss of appetite. Therefore, it was necessary to repeatedly apply dose reductions. First, with the schedule of 5 days ON (60 mg) and 2 days OFF, later with the dosage of 40 mg/die, and, finally, another period with a schedule of 5 days ON (40 mg) and 2 days OFF. The last strategy was well tolerated and was continued.

In December 2023, the follow-up CT scan documented a numerical and dimensional reduction in the metastases. Therefore, Cabozantinib was continued, and the subsequent CT scans showed stable disease.

**Figure 1:**
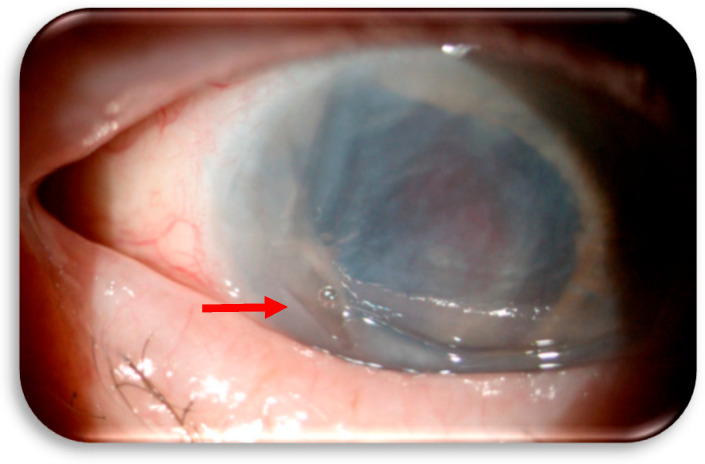
The red arrow indicates the marginal corneal ulcer with perforation.

In December 2024 (at 15 months from Cabozantinib commencement), the patient began to experience pain and vision loss in the right eye, so he was treated at the Ophthalmology Unit of “S. Croce and Carle” Hospital. The patient had no history of ocular disease, but he started to suffer from a marginal corneal ulcer in the right eye. The most common causes of corneal ulceration are contact trauma, damaging agents (chemical agents and prolonged use of drugs such as topical NSAIDs), infections, or dry eye. However, all of these causes were excluded in our patient. Topical therapy with ophthalmic ointment containing chloramphenicol 0.4%, colistin, and tetracycline 0.4% (Colbiocin, Sifi Spa, Catania, Italy) was initiated and continued for one month until the ulcer re-epithelialized. The dose and administration scheme of Cabozantinib were not modified. However, in January 2025, the patient returned to the Ophthalmology Unit before the scheduled follow-up visit due to worsening of the clinical condition. Recurrence of the ulcer was identified, this time with perforation of the cornea (Figure 1). Cabozantinib was suspended, a therapeutic contact lens was immediately fitted, and topical therapies with moxifloxacin 0.5%, netilmicin 0.3%, and atropine 1% drops were initiated.

**Figure 2:**
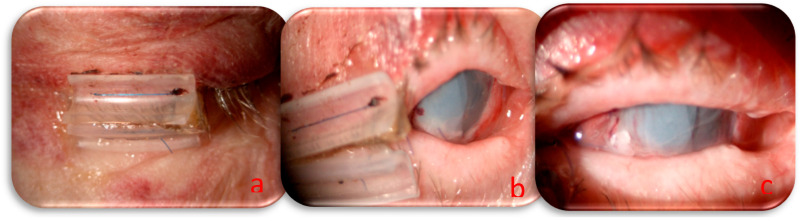
(**a**) Lateral tarsorrhaphy with 6/0 Prolene suture and silicon hose; (**b**) eye in the primary position with lateral tarsorrhaphy and the cornea covered by triple HAM layers; (**c**) eye facing left to better visualize the perforated area covered by the eyelid.

Based on the systemic condition of the patient and the necessity to continue oncological treatment, we decided to perform urgent surgery and a combined approach with a human amniotic membrane (HAM) to close the corneal perforation and lateral tarsorrhaphy to reduce ocular exposure and enhance the healing process. The HAM was sutured to the limbus using a 10/0 nylon suture to guarantee perfect corneal closure, and a triple layer was used to increase the permanence on the perforated area. The tarsorrhaphy was made with absorbable 6/0 sutures (Vicryl, Ethicon, Johnson & Johnson, Somerville, MA, USA) on the tarsal plate and non-absorbable 6/0 sutures with a silicon hose (Vicryl, Ethicon, Johnson & Johnson, Somerville, USA) on the anterior eyelid margin; the use of a mixed suture allowed us to regulate the timing and grade of eyelid aperture during recovery. The day following surgery, residual visual acuity was assessed based on hand motion, amniotic membranes were appropriately positioned, and the anterior chamber was filled with aqueous humor, indicating successful closure of the corneal perforation (Figure 2). Postoperatively, a topical regimen comprising moxifloxacin 0.5% (Vigamox, Alcon, Fort Worth, TX, USA), netilmicin 0.3% (Nettacin, Sifi spa, Catania, Italy), atropine 1%, and ocular lubricants was established.

**Figure 3:**
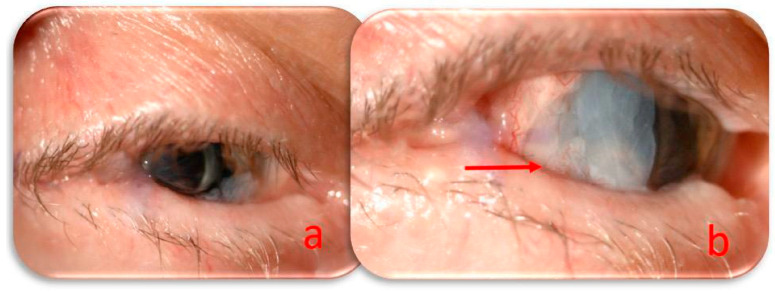
(**a**) The eye in the primary position with lateral tarsorrhaphy covering the site of ulceration; the central cornea cleared after HAM absorption; (**b**) the eye facing left to better visualize the perforated area (red arrow) covered by the residual HAM and conjunctiva.

At the monthly follow-up, the lateral tarsorrhaphy was partially opened by removing the non-absorbable suture. The perforation area was still covered with an amniotic membrane and a conjunctival flap. The central cornea appeared clear after HAM absorption, the anterior chamber had regular depth, the iris was dysmorphic, the scleral fixed intraocular lens was correctly positioned, and the intraocular pressure was 14 mmHg. The visual acuity to count fingers was compromised due to severe macular atrophy preceding the corneal perforation (Figure 3). The treatment regimen was modified with intensive ocular lubrication, and follow-up visits were scheduled every two weeks.

Unfortunately, our patient’s clinical condition deteriorated rapidly, probably due to the progression of the disease, culminating in his death.

## 4. Discussion

Cabozantinib is a drug belonging to the VEGFR-TKI family that has proven to be effective in the treatment of various advanced-stage neoplasms. Multiple studies on Cabozantinib have reported a range of AEs—some very common, while others are relatively rare—largely similar to those observed with other VEGFR-TKIs. The most frequent ones include diarrhea, fatigue, hypertension, HFS, and hypothyroidism, whereas the less common ones include thromboembolic or hemorrhagic events that can lead to severe complications [15]. Very few data in the literature concern VEGFR-TKIs-mediated ocular AEs, and these are limited to a handful of case reports describing bilateral optic disc edema, exudative retinal detachment, and choroiditis [8,9,11]. No data have been published about corneal perforation during Cabozantinib therapy. To date, only one case of this AE has been reported in connection with another VEGFR-TKI, Regorafenib [10]. Other targeted drugs can also cause corneal diseases, as reported in some case reports. The FGFR-TKI infigratinib can cause dry eye, severe punctate keratitis, and recurrent corneal ulcers [16]. EGFR inhibitors such as cetuximab and erlotinib have been reported to cause ocular adverse reactions, such as persistent corneal epithelial defects, corneal lysis, and perforation [17,18]. Anlotinib has many targets, and its mechanism of causing keratopathy may be similar to other TKI drugs [19].

Given the limited knowledge about these AEs, we deemed it necessary to describe another case of corneal perforation in a patient affected by advanced RCC treated with Cabozantinib. Our patient received Cabozantinib after progression to Pembrolizumab plus Axitinib, according to the results of the METEOR trial [5]. The patient started therapy at a dosage of 60 mg/die, although it was necessary to apply some dose reductions because of some grade 2 AEs (according to CTCAE v6.0), such as asthenia, diarrhea, dysgeusia, and loss of appetite. However, after approximately 15 months of treatment, the patient began to experience pain and vision loss in the right eye. Thus, with the diagnosis of corneal perforation, medical and surgical treatment were performed. The most common causes of corneal ulceration are contact trauma, damaging agents (chemical agents, prolonged use of drugs such as topical NSAIDs), infections, or dry eye. However, all of these causes were excluded in our patient. Based on the very limited data about ocular AEs on VEGFR-TKIs, there is no clear evidence to directly associate corneal perforation with Cabozantinib. However, several factors might contribute to explaining this correlation. First of all, similar to the other VEGFR-TKIs, Cabozantinib leads to the block of neoangiogenesis through the inhibition of VEGFR2 [11,12,13]. The result is the reduction in nutrient and oxygen supply to the tumor, thereby slowing its growth. Moreover, this condition favors the occurrence of a hypoxic TME, leading to a decrease in the release of cytokines and growth factors, rendering the microenvironment less favorable to tumor growth. On the other hand, it is well known that various growth factor receptors, including VEGFR, FGFR, and PDGFR, are expressed in normal ocular tissues such as corneal stroma, corneal epithelium, and conjunctival endothelium [20]. The stimulation of these receptors by the corresponding growth factor favors vascular permeability and corneal cell proliferation, enabling corneal homeostasis and recovery [20]. Although the cornea is not vascularized, it is nourished by the diffusion of substances from adjacent vascular structures through the tear film [21]. Therefore, these different mechanisms of action by VEGFR-TKIs might undermine the ocular environment due to a lack or imbalance of growth factors in the tear film, with a reduction in corneal epithelium proliferation. This condition might cause the occurrence of dry eye and a delay in corneal healing. Moreover, VEGFR is also expressed on corneal nerves, so VEGF acts as an important neurotrophic growth factor [5]. In this regard, the local administration of anti-VEGF therapy determines a reduction in corneal nerve fiber density, the number of fibers, the number of bifurcations, and the total nerve fiber length [22]. Therefore, this might lead to an increase in dry eye syndrome and even the onset of corneal ulcers. However, on the other hand, one study demonstrated the effectiveness of ocular therapy with anti-VEGF agents in blocking neovascularization without ocular toxicity [23,24]. Nevertheless, these considerations are hypotheses not supported by any in vivo or in vitro evidence. Therefore, it is not actually possible to define a sure correlation between corneal perforation and the drug. Moreover, to date, no reliable predictive factors for VEGFR-TKI-mediated ocular toxicity can be defined due to the few data available. However, the clinical experience with our patient can provide important information about the management of patients treated with Cabozantinib and VEGFR-TKIs in general. In detail, patients undergoing treatment with these drugs should be closely monitored to enable the early identification of potential ocular AEs. Management of corneal perforation should include the administration of topical antibiotics to prevent secondary infections, the employment of medications that promote corneal re-epithelialization, corneal tarsorrhaphy, and the suspension of VEGFR-TKI treatment until healing occurs. In cases of persistence or recurrence, permanent discontinuation of therapy should be considered.

## 5. Conclusions

Given the very limited data regarding ocular AEs under VEGFR-TKIs, we felt it was necessary to inform the scientific community about another case of corneal perforation in a patient affected by advanced RCC on therapy with a VEGFR-TKI, Cabozantinib.

Based on Cabozantinib’s mechanisms of action, it is possible to suppose a correlation with this side effect. Particular importance should be placed on ophthalmologic surveillance during this type of therapy, in case of ocular symptoms.

Further in vitro and in vivo studies are necessary to deepen knowledge about VEGFR-TKI-mediated ocular AEs, also through the development of scoring scales to assess the correlation between drugs and patients’ ocular symptoms.

## Data Availability

The original contributions presented in this study are included in the article.
